# Perception of pediatric residents from a tertiary hospital in the city of México regarding their training during the COVID-19 pandemic

**DOI:** 10.1186/s12909-022-03776-y

**Published:** 2022-10-17

**Authors:** Eduardo Bracho Blanchet, Miguel Künder Klünder, José Antonio Orozco Morales, Carolina Hill De Titto, Diana Avila Montiel

**Affiliations:** 1 Directorate of Research, Hospital Infantil de México Federico Gómez, CDMX México, Calle Dr. Márquez 162, Col. Doctores, CP 06720 Cuauhtémoc, Mexico; 2 Directorate of Education, Hospital Infantil de México Federico Gómez, CDMX México, Calle Dr. Márquez 162, Col. Doctores, CP 06720 Cuauhtémoc, Mexico

**Keywords:** COVID-19, Medical education, Online education

## Abstract

**Backgrounds:**

On March 11, 2020, the World Health Organization (WHO) declared the novel coronavirus (COVID-19) outbreak a global pandemic, which changed the residents’ teaching and learning process. The purpose of this study was to determine residents’ satisfaction and impressions on their training during the pandemic in a tertiary pediatric hospital.

**Methods:**

This was a descriptive cross-sectional study. An online survey was designed to determine residents’ demographic and personal characteristics, as well as their perception about the theoretical and practical training, as well as about their emotional situation. The analysis separated medical students from surgical students in order to identify any differences existing between these groups, for which χ2 was calculated.

**Results:**

Overall, 148 of 171 residents (86.5%) responded to the questionnaire; 75% belonged to the medical specialty and 25% to the surgical specialty. Statistically significant differences were found in terms of those training aspects they were concerned about during the pandemic (p < 0.001) and about the difficulties associated with online learning (p = 0.001). Differences were also found regarding their satisfaction toward the time needed to complete their thesis (p = 0.059) and activities outside the hospital (p = 0.029). Regarding their degree of satisfaction in general, most medical specialty students felt slightly satisfied (43.2%) and surgical specialty students felt mostly neutral (37.8%). Regarding their feelings about their mental health, statistically significant differences were found between both groups (p = 0.038) although both groups reported the same percentage of overall dissatisfaction (2.7%) in this area.

**Conclusion:**

The COVID-19 pandemic has brought significant challenges to medical education systems. Lack of practice in decision-making and maneuver execution are concerns for residents and may affect their future professional performance.

## Introduction

On March 11, 2020, the World Health Organization (WHO) declared the novel coronavirus (COVID-19) outbreak, originally described in Wuhan, in the Chinese province of Hubei, a pandemic. Since that date, the general belief was that this new disease would put the society and health systems to the test [1].

In México, the first imported cases were described on February 28 and local transmission was confirmed on March 24, 2020 [2].

It soon became clear that this disease is highly contagious, and that the exponential increase of infections caused hospital saturation and resource scarcity in terms of staff and medical equipment, as well as shortage of intensive care beds and ventilators. In order to tackle these problems, hospital reconversion, that is, the process by which different hospitals prepare for the care of patients during a health crisis (in this case, the one caused by COVID-19), was established in China originally and later on in Italy, the United States of America and in our own country. Hospital reconversion involves postponing outpatient specialty consultation, elective surgery, auxiliary diagnostic tests, physical medicine, and group psychological consultation, among other measures [3].

On March 17, 2020, the Association of American Medical Colleges recommended the suspension of all activities involving direct contact between patients and medical students due to the COVID-19 pandemic [4]. The need to reduce hospital overcrowding led to a decrease in the number of residents at their teaching sites [5], probably affecting residents in their final years of specialty the most [6].

In our country, the health system had to face the pandemic containment by making use of all available resources, both material and human. Our Institute was reconverted into a pediatric COVID-19 hospital, which implied the redistribution of residents to services related with the care of COVID-19 patients, with the consequent modification of their academic activities [7]. Among the academic activities implemented during the pandemic, online learning or e-learning stands out.

The definition of online learning may vary according to different organizations, but in essence, it is the use of electronic media for training and education, using the web, computers, and virtual classrooms, and developing digital content [8].

Teaching processes vary according to the type of specialty, but all of them require the acquisition of theoretical and practical skills. For this reason, exploring the current training methods worldwide, which are mainly based on online learning, is now of the utmost importance [9]. In addition to exploring how effective this learning is in comparison with traditional learning, studying its influence on the perception and satisfaction of future health professionals becomes all the more necessary [10].

Learning satisfaction could be defining as the student’s feelings and attitudes towards the education process and the perceived level of fulfillment connected to the desire to learn [11].

Satisfaction has been related to successful educational processes. It involves the expectation that students have about their learning and the quality of the perceived education service received. Exploring satisfaction is a good indicator for institutions on the effectiveness of educational programs. [12–14].

Since early 2020, concerns regarding residents’ education began to be published, as well as strategies that should be adopted in an effort to alleviate these deficiencies [15–18]. Subsequently, several reports were published on some results about the strategies adopted during the pandemic. However, most of them referred to the reduction of clinical activities, especially in surgical specialties [19] such as general surgery [20], neurosurgery [21], otorhinolaryngology [22], gynecology-obstetrics [23], urology [24], orthopedics-trauma [25], and interventional cardiology [26], in which emphasis is placed on the reduction or suspension of elective surgeries, visits, or outpatient consultations. Reports on the impact of the pandemic on medical specialties were scarce and, as in surgical specialties, confirmed a reduction in the clinical training or in the strategies adopted, for example, in radiology [27, 28], dermatology [29], internal medicine [30] and gastroenterology [31]. Regarding residency in pediatrics or pediatric specialties, there are few publications on training and learning management in the face of the pandemic [32], or on the creation of a file of previous conferences [33].

Although there are reports on the pandemic’s impact on residents, most of them refer to surgical specialties [5, 34, 35], and there are few reports on medical specialties [36]. The impact on pediatric residency or its subspecialties has been scarcely described [37, 38]. In this regard, this lack of evidence points out to the fact that undergraduate students are satisfied with online learning, being the preclinical years the most benefited period, in contrast to students in clinical stages [39].

There is concern in the world about the development of the future health professionals trained in this era, whose clinical training time has been reduced and replaced by online training [40, 41]. Therefore, the aim of this study was to determine the level of satisfaction with theoretical and practical learning, as well as the emotional well-being, of residents from a pediatric hospital converted into a COVID-19 hospital, and to analyze the differences between residents of medical specialties (MRs) and those of surgical specialties (SRs).

In Mexico residents have the degree of graduated general practitioners however, being residents of some specialty or medical or surgical subspecialty means that they have decided to continue their training so in the hospital they are seen as students.

In the specialization process known as residency, doctors are seen as students. They obey a hierarchy given by the degree of residence they are studying (it can range from two to seven years, depending on the specialty they have chosen) and must follow instructions from their professors, which are doctors that work professionally in the hospital.

In order to obtain the degree of specialist, residents must submit a thesis related to their area of specialty. The thesis consists of a clinical research protocol, which can be from the design and implementation of an observational or even experimental study.

The teaching-learning methodology has focused on problem-solving with the aim that residents form habits and skills to reason critically and reflexively about health problems. In this sense, theoretical-practical training is complemented by visits to other hospitals, attendance at seminars and congresses, and presentation of clinical cases.

During the pandemia, online activities were implemented. The classes were online via zoom and were complemented by seminars and presentations of clinical cases online.

Although students have different tutors and managers (doctors that work in the hospital), according to the area of specialty they have chosen, there is a coordination team in the Teaching Directorate, which is responsible for monitoring the educational programs of each area, as well as supporting students with any academic or administrative issue related to their training.

## Methods

An online survey was conducted based on literature search, which was conducted from January 2020 to March 2021 in MEDLINE, using the following MeSH terms and keywords: education, programs, medical education, medical, students, learning, e-learning, COVID-19, and SARS-CoV-2 and with filters such as human studies and indexed studies. Additionally, we searched Google Scholar and the reference lists of the articles found to identify other relevant studies. Four study were included to select the surveys items. [42–45]

The survey was elaborated and applied using Google Forms and covered 4 main areas as follows: residents’ demographic and personal characteristics, perception of theoretical training, perception of practical training, and perception of their own emotional situation.

The survey included 42 items: 13 were designed to characterize the students, 14 to explore their satisfaction with theoretical training, 11 to explore their satisfaction with practical training and 4 to explore their satisfaction with their emotional state. The survey included items combining dichotomous, 5 points Likert-scale, and multiple-choice responses.

The answers to the 5 points Likert scale were categorically expressed with text (Not at all satisfied, slightly satisfied, neutral, very satisfied, extremely satisfied) to explore the resident´s perception of their learning process.

In addition, the questionnaire included an initial section with an informed consent form which residents had to fill out in order to be eligible to complete the survey. No validation process was performed for this survey. The protocol was submitted to the Research Committee of the hospital under authorization no. HIM-SR-2021-004.

The survey was sent through the Teaching Department via e-mail to all pediatric and pediatric specialty residents who had completed any year of their residency during March 2020 to February 2021.

The outcomes were analyzed using SPSS and Stata software. Descriptive statistics were used as percentages and a comparison was made between residents of medical and surgical specialties to identify differences between both groups, for which the p-value was calculated with Fisher’s exact test and to compare the variables of the Likert scale, χ2 was calculated.

## Results

A total of 171 applications were sent and 148 residents (86.5%) responded: 111 (75%) belonged to medical specialties and 37 (25%) to surgical specialties, with 80.2% female participants and 19.8% male paticipants. In relation to age, the majority of participants (60.8%) were 25 to 30 years old. 88.5% of the residents were single. Regarding the year of specialty, 27.7% were in their first year, 14.2% in their second year, 14.9% in their third year and 43.2% were in a subspecialty (Table 1).

**Table 1:** Sociodemographic characteristics of the residents of Hospital Infantil de México Federico Gómez (HIMFG)

	Medical Specialties n = 111 (75%) %(n)	Surgical Specialties n = 37 (25%) %(n)
**Sex**		
Female	80.2 (89)	59.5 (22)
Male	19.8 (22)	40.5 (15)
**Age Range**		
25 to 30 years	67.6 (75)	40.5 (15)
31 to 35 years	30.6 (34)	51.4 (19)
36 to 40 years	1.8 (2)	8.1 (3)
**Marital status**		
Single	89.2 (99)	70.3 (26)
Married or free union	10.8 (12)	29.7 (11)
**Year of specialty**		
First	33.3 (37)	10.8 (4)
Second	16.2 (18)	8.1 (3)
Third	18.9 (21)	2.7 (1)
Subspecialty	31.5 (35)	78.3 (29)

In terms of online learning during the pandemic, residents were asked 6 questions.

Initially, they were asked whether they were familiar with this type of learning, finding that most of the students were not (n = 106). Regarding the type of online activities they performed, they reported that live lessons were the main activity (MRs = 85.6% and SRs = 70.3%), and regarding the importance of this method for their training, most of them described it as important (MRs = 94.6% and SRs = 83.8%). It is worth mentioning that no differences were found between MRs and SRs for these factors.

However, statistically significant differences were found in two items associated with the residents’ concerns regarding their preparedness for the pandemic (p ≤ **0.001**), finding that the main concern for MRs was their preparedness for clinical decision-making, while, for SRs, the main reason for concern was their preparedness to treat patients (Table 2).

Likewise, MRs reported greater difficulties (64%) with this type of learning than SRs (36%) (p ≤ **0.001**), with technical failures being the most common difficulties.

**Table 2:** Online learning (N = 148)

	**MRs** **n 111** **% (n)**	**SRs** **n = 37** **% (n)**			**P-value**
**They are familiar with platforms (Zoom, Data Webinar, Google classroom…)**					
Yes	30.6 (34)	21.6 (8)			
No	69.4 (77)	78.4 (29)			0.400
**Types of online academic activities**					
Live online lessons	85.6 (95)	70.3 (26)			
Webinars	8.1 (9)	13.5 (5)			
Individual counseling	3.6 (4)	8.1 (3)			0.122
Others	2.7 (3)	8.1 (3)			
**They consider e-learning an important part of their training**					
Yes	94.6 (105)	83.8 (31)			
No	5.4 (6)	16.2 (6)			0.074
**Aspects of concern regarding their preparedness for the pandemic**					
Clinical decisions	61.3 (68)	10.8 (4)			
Diagnosis	8.1 (9)	8.1 (3)			
Treatment	18.0 (20)	70.3 (26)			**< 0.001**
Others (Lack of practical and surgical skills, lack of experience)	12.6 (14)	10.8 (4)			
**They experienced difficulties associated with online learning**					
Yes	64 (71)	32.4 (12)			**< 0.001**
No	36 (40)	67.6 (25)			
**Main difficulties**					
Distraction	25.2 (28)	13.5 (5)			
Boredom	9 (10)	8.1 (3)			
Bad lecturers	2.7 (3)	0 (0)			
Technical failures (connection, equipment, etc.)	30.6 (34)	18.9 (7)			0.093
Poor audiovisual material	2.7 (3)	2.7 (1)			
No difficulties	29.7 (33)	56.8 (21)			

As for the degree of satisfaction with their current training process when compared to the pre-pandemic stage, it was similar (p = 0.511) between MRs and SRs; the majority of residents being slightly satisfied (SRs 37.8%) and not at all satisfied (MRs 43.2%) with their current training.

**Fig. 1:**
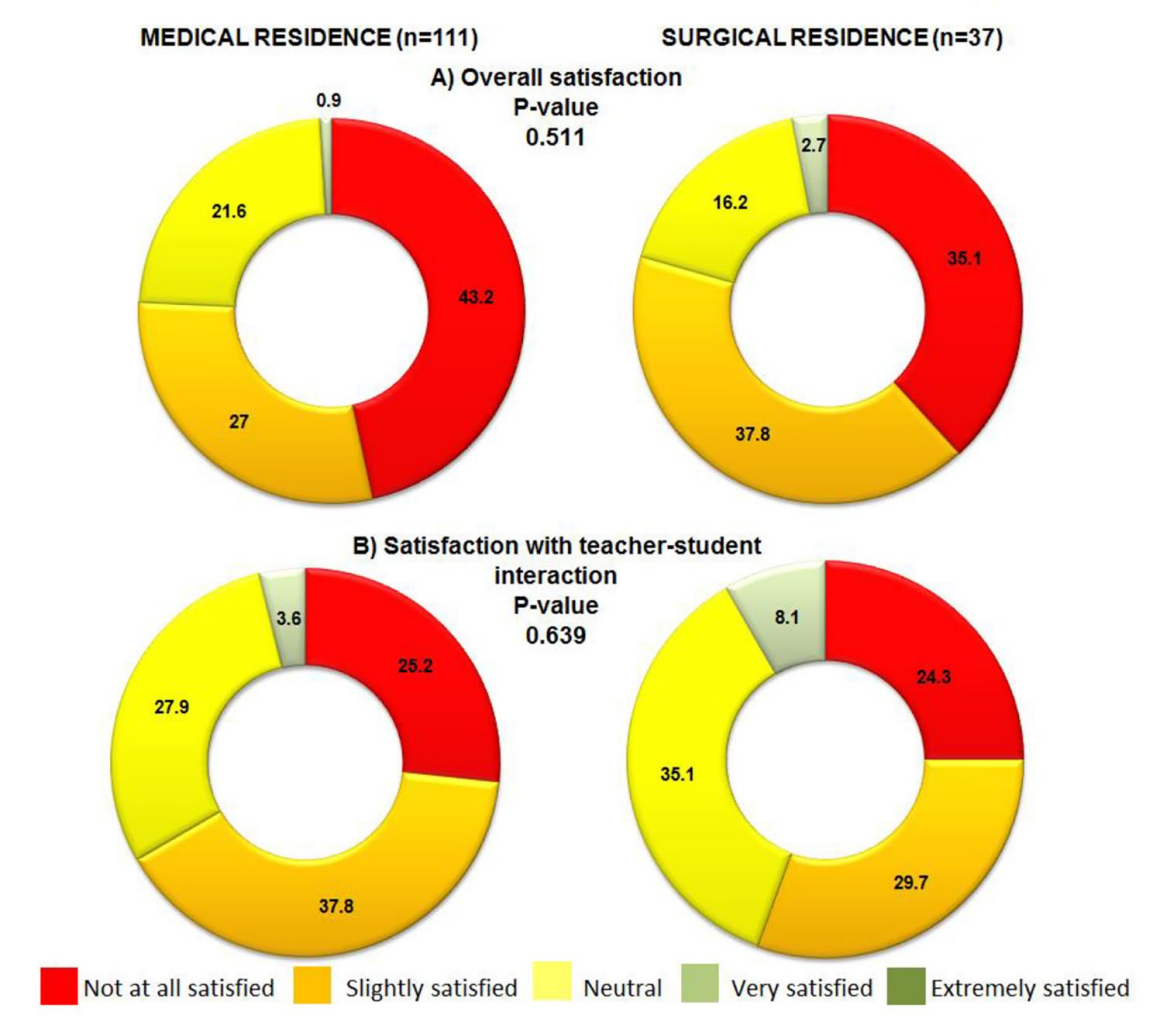
Traditional learning vs. Online learning

The degree of satisfaction with the interaction they had with their teachers was similar in both specialties (p = 0.639), although the majority (35.1%) of SRs indicated being very satisfied, while the majority of MRs (37.8%) were neutral (Fig. 1).

Regarding theoretical training, residents were asked how they felt about the academic activities organized by the teaching management, and the results in both types of specialties were mainly neutral (p = 0.697).

Regarding satisfaction with the academic activities carried out in their departments, SRs were twice as satisfied as MRs (21.6% vs. 10.8%); however, no statistically significant differences were found (p = 0.327). Regarding satisfaction with activities outside the hospital, it was statistically significant lower in MRs than in SRs (p = 0.020).

**Fig. 2:**
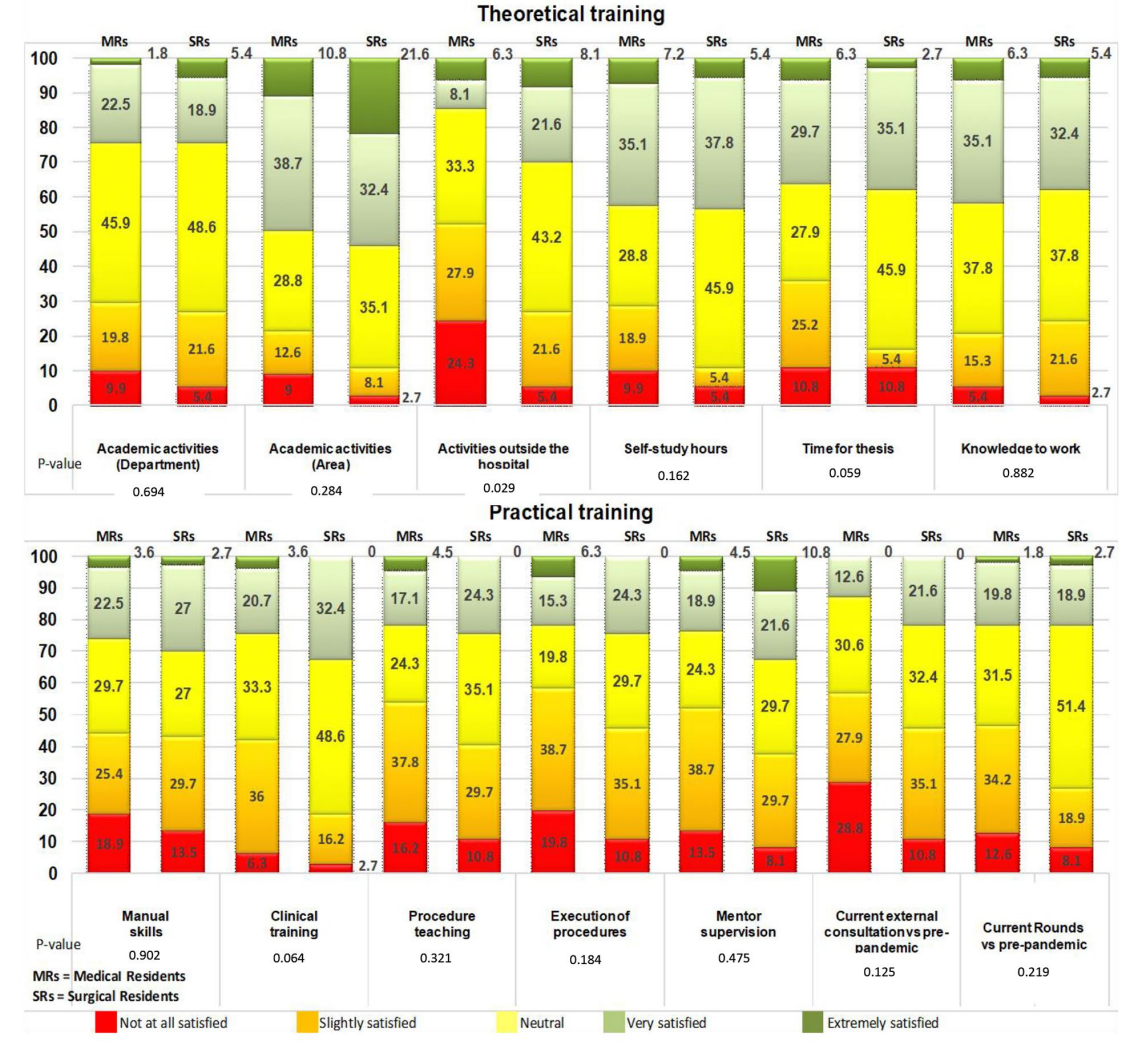
Percentage of satisfaction of medical (n = 111) and surgical (n = 37) residents with their training

In terms of self-study hours, there were no statistically significant differences between the groups; however, with regards to the time they could allocate for their thesis, statistically significant differences were found (p = 0.059). While both groups reported feeling equally dissatisfied, most of the MRs indicated feeling slightly satisfied (25.2%) and neutral (27.9%), while SRs felt more neutral (45.9%) and satisfied (35.1%). When asked about their perception regarding their knowledge to face the labor world, both groups reported to feel mostly neutral (Fig. 2).

Regarding for their practical training, when asked about their satisfaction with their manual skills, MRs were more neutral, and most SRs were slightly satisfied. It is worth mentioning that no student in either group reported feeling totally satisfied. In terms of their clinical training, MRs were less satisfied compared to SRs. Finally, regarding procedure teaching, most MRs referred to themselves as being slightly satisfied and the SRs as neutral. However, 4.5% of MRs were extremely satisfied while no SRs were extremely satisfied.

In relation to the execution of procedures, most MRs and SRs were slightly satisfied (38.7% and 35.1% respectively), and although 6.3% of MRs were extremely satisfied versus no SRs, also in this item, MRs reported a higher degree of dissatisfaction (p = 0.184). Regarding mentor supervision, SRs reported higher satisfaction than MRs, with no statistically significant differences.

Comparing current external consultations versus pre-pandemic consultations, neither group was completely satisfied; 28.8% MRs were not at all satisfied, representing a 10% more than SRs. Regarding current hospital rounds versus pre-pandemic hospital rounds, there was less satisfaction among MRs. No statistically significant differences were found between groups on any of the items (Fig. 2).

**Fig. 3:**
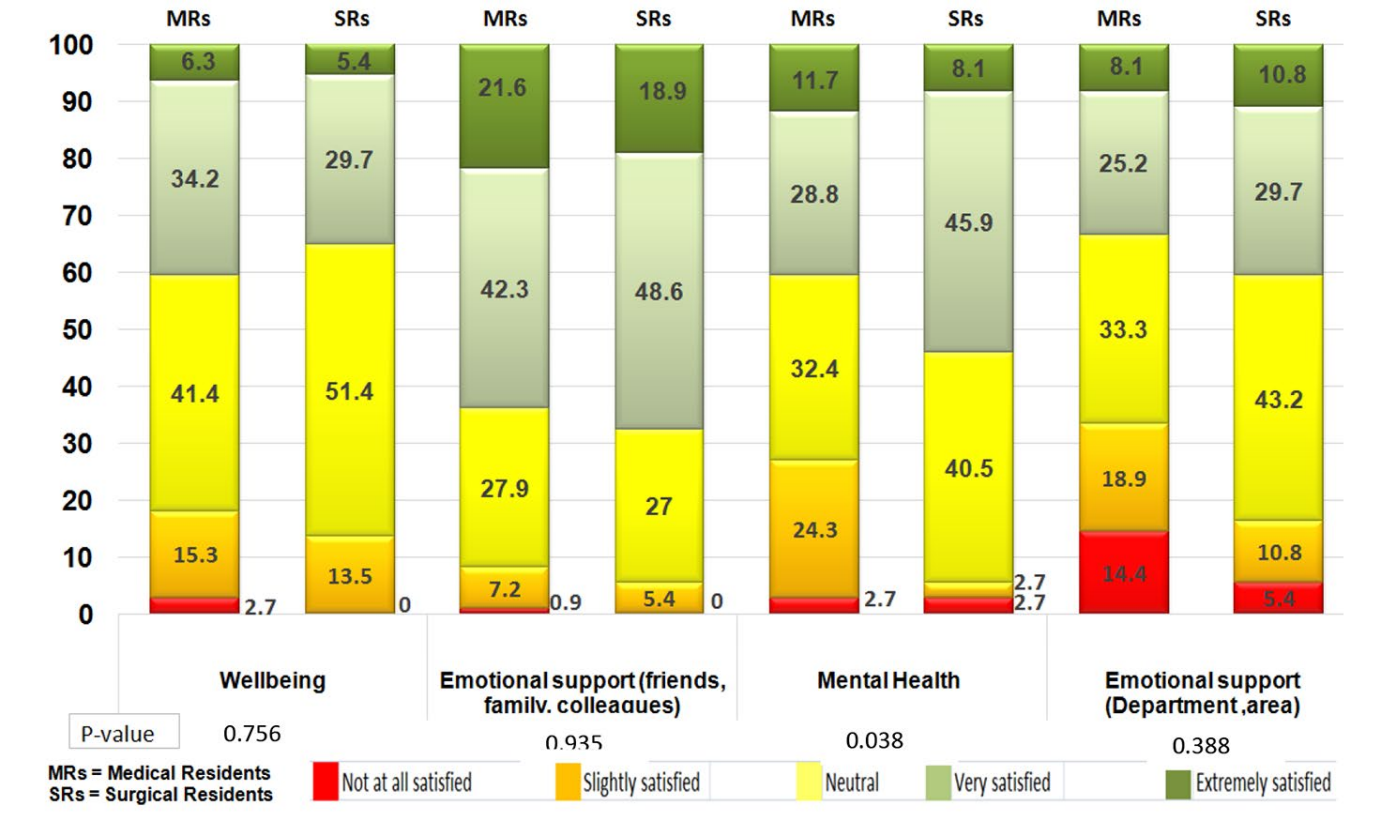
Percentage of satisfaction with the emotional area (Medical (n = 111) and Surgical (n = 37) residents)

Finally, Fig. 3 depicts the results of the items aimed at exploring the emotional area of MRs and SRs. When asked about their degree of satisfaction with their well-being, a 2.7% of MRs felt not at all satisfied, with no SRs in this item, and the majority of residents felt neutral.

When asked regarding emotional support from friends, family, and colleagues, the majority of residents felt very satisfied.

When exploring how they felt about their mental health, although 2.7% in both groups expressed feeling not at all satisfied in this area, statistically significant differences were found between the groups (p = 0.038), with a greater number of satisfied SRs against dissatisfied MRs.

In terms of the emotional support received from their department, MRs reported feeling less satisfied compared to SRs.

## Discussion

Due to the COVID-19 pandemic confinement, educational activities were disrupted at all levels. Hospital reconversion has affected care and teaching activities, with the subsequent impact on resident training.

Slightly more than half of the residents (56.7%) were studying pediatrics and the rest were studying a subspecialty. A total of 75% were studying medical specialties or subspecialties and 25% surgical specialties, which makes our population a mixture of both disciplines and allowed us to compare some results between the two of them.

However, none of these reports refer to residents’ self-perception about their training, both theoretical and practical, during the first year of the pandemic. This was the objective of our study which is, to our knowledge, the first to report residents’ perception of the impact of the pandemic on their training process as pediatricians or pediatric specialists. Our survey was answered by 86.5% of the 171 residents, and therefore, it adequately reflects the perceptions in an exclusively pediatric hospital converted into a COVID hospital. Health care and academic activities changed drastically; outpatient consultations, hospitalizations, and elective surgeries were cancelled; face-to-face patient care was reduced and many of the residents had to care for COVID-19 patients; and external rotations and congresses were cancelled. Almost all academic sessions were delivered online despite the fact that 71.6% of our residents were not familiar with distance learning platforms. However, it is important to start considering online learning a formal didactic resource, since, as indicated by Mian et al., it has proven to be of great help for theoretical training and allows continuing education even in emergency situations [46].

As in the reports by Tapper J et al., the residents in this study reported connectivity issues and difficulties accessing the platforms as the main limiting factors to continue their training. This difficulty was reported by 56% of our residents, especially MRs (64% vs. 32% SRs, *p = 0.001*), with technical failure being the predominant factor in 27%, half of the number reported by Dasgupta et al. with regards to a group of ophthalmology residents [47]. This is probably related to the fact that, in Latin America, only 1 in 2 households has broadband Internet connection [48], and therefore, appropriate resources and conditions for online studying should be implemented [49, 50]. Other difficulties were distraction during lessons (22.2%) and boredom (8.7%), which is consistent with what was published for emergency and internal medicine residents, who were more engaged in other activities such as literature searches, answering e-mails, and even exercising during online lectures. Engaging the attention of learners is critical in online education. In this regard, some options include asking group questions via social networks, conducting small group sessions, and even having more game-like activities [51].

It is striking that practically half of our residents reported being slightly or not at all satisfied with their current training compared to the pre-pandemic period (Fig. 1), as has been reported in orthopedic residents [52]. In this regard, 89.7% of the residents surveyed by Guo et al. think that their education was negatively affected by the pandemic [22]. On the contrary, with regards to webinars, neurosurgery residents think that they are more useful than face-to-face conferences [53] and half of the ophthalmology residents surveyed think that they should continue in the post-pandemic stage [49]. One of the most important concerns expressed by the MRs in our study was related with making clinical decisions, whereas the SRs expressed greater concern about their treatment-oriented training (70.3%). This is consistent with other reports [22, 54], perhaps the surgical nature of their specialty, make SR residents more focused on such treatments.

When evaluating residents’ perception of their theoretical training, about 50% were satisfied with the academic activities, but MRs were significant more satisfied than SRs with out-of-hospital activities such as conferences or webinars. This is probably related to the fact that out-of-hospital activities of SRs are often associated with the execution of other surgical procedures in the out-of-hospital rotations that were cancelled. Regarding the time available for thesis preparation, MRs were significantly less satisfied than SRs, perhaps because they had more out-of-hospital activities. Regarding time for study, most of our residents reported being very satisfied (35.1% of MRs) or neutral (45.9% of SRs), contrary to what was reported by orthopedic and trauma residents, who reported a 46% restriction in time for self-study [25].

In relation to practical training, high levels of dissatisfaction were predominant, generally above 40%, with MRs being less satisfied and with no statistically significant differences with respect to SRs. Many studies reported a decrease in practical activities in surgical specialties [20, 24, 26, 55, 56], but our results prove that residents in predominantly medical specialties, such as pediatrics and its associated medical specialties, were also affected in terms of practical learning. We must explore new methods of academic training and, in this regard, social networking is a tool that may have been underutilized [19], although it is undoubtedly on the rise as a result of the pandemic [57]. Other strategies that abound in practical training include files of radiological studies or clinical cases that can be constantly consulted [33], the use of tele-dermatology [29] or surgical simulators.

Regarding satisfaction with their emotional well-being and mental health, it is to be noted that 89.5% of the total number of residents reported having cared for patients with COVID-19, of whom 27.7% got infected. Facing a new disease with less medical knowledge, having to wear special protective equipment, managing a high patient load, facing life-and-death decisions and desires, very often through video calls, is a source of psychological stress [30]. A commonly reported fact is the fear of residents getting the disease themselves [30, 58–60], which was corroborated in our study, since 50.7% of the residents were constantly afraid of getting the disease and 26.4% felt occasional fear.

This undoubtedly has an impact on residents’ mental health. Other studies have reported conditions such as anxiety about their future [23], mental health alterations [21], decreased quality of life [52] and burnout [61]. In this study, MRs were more frequently slightly or not at all satisfied with their mental health compared to SRs (27% vs. 5.4%, *p = 0.038*). This is something that should be confirmed in other series comparing MRs with SRs and, if confirmed, the cause should be investigated in greater depth. Some possible strategies could be that directors of residency programs in pediatrics, and probably other specialties, would consider reducing extremely long on-call shifts and changing to shorter schedules followed by rest, at least while the pandemic contingency is maintained [62].

## Limitations

Although our study adds to the knowledge on pediatric residents’ perception about the impact of the COVID-19 pandemic on their education, it has some limitations. Data are based on self-reports, so they are subjective and should be interpreted with caution. This paper did not investigate their academic and professional performance or the relationship with the subject matter of the study, however interesting this could be, as that was not the purpose of this research. One potential variable is the effect of the pandemic *per se* on residents’ perception, bearing in mind that, when this research was carried out, our country was immersed in the second wave of contagion, and vaccination against SARS-Cov-2 had just commenced.

Regarding their wellbeing, it´s possible students are not aware of these aspects, so they reported as neutral, contrary reported in the literature, in this sense, there is a methodological limitation by not having used a validated scale to know the emotional aspects of the students.

Finally, as in the rest of the world, this pandemic has brought significant challenges to medical education systems, and, as in many other areas, the true long-term impact on physician training still remains unknown. Medical education systems should promote the use of technologies in their educational curricula and find further technologies and strategies that allow students to continue their intellectual training and personal development even in times of adversity. The results of this study can be used as a basis for the generation of personal and material resource organization and management strategies, both, of health institutions in order to meet the needs expressed by residents.

## Conclusion

This study shows residents’ perception about their training during the COVID-19 pandemic. It also reveals that, as a result of the pandemic, residents’ uncertainty when it comes to making clinical decisions or residents’ lack of clinical practice could affect their performance in the labor world. Likewise, it shows that residents of medical and surgical specialties have different degrees of satisfaction regarding their training and emotional aspects.

## Data Availability

Data are available in Excel format upon request. To request the material please contact the corresponding author, Diana Avila Montiel.
